# A Free-Knot Spline Modeling Framework for Piecewise Linear Logistic Regression in Complex Samples with Body Mass Index and Mortality as an Example

**DOI:** 10.3389/fnut.2014.00016

**Published:** 2014-09-29

**Authors:** Scott W. Keith, David B. Allison

**Affiliations:** ^1^Department of Pharmacology and Experimental Therapeutics, Division of Biostatistics, Thomas Jefferson University, Philadelphia, PA, USA; ^2^Department of Biostatistics, Office of Energetics and Nutrition Obesity Research Center, University of Alabama at Birmingham, Birmingham, AL, USA

**Keywords:** free-knot splines, non-linear modeling, logistic regression, bootstrap, complex samples, body mass index

## Abstract

This paper details the design, evaluation, and implementation of a framework for detecting and modeling non-linearity between a binary outcome and a continuous predictor variable adjusted for covariates in complex samples. The framework provides familiar-looking parameterizations of output in terms of linear slope coefficients and odds ratios. Estimation methods focus on maximum likelihood optimization of piecewise linear free-knot splines formulated as B-splines. Correctly specifying the optimal number and positions of the knots improves the model, but is marked by computational intensity and numerical instability. Our inference methods utilize both parametric and non-parametric bootstrapping. Unlike other non-linear modeling packages, this framework is designed to incorporate multistage survey sample designs common to nationally representative datasets. We illustrate the approach and evaluate its performance in specifying the correct number of knots under various conditions with an example using body mass index (BMI, kg/m^2^) and the complex multistage sampling design from the Third National Health and Nutrition Examination Survey to simulate binary mortality outcomes data having realistic non-linear sample-weighted risk associations with BMI. BMI and mortality data provide a particularly apt example and area of application since BMI is commonly recorded in large health surveys with complex designs, often categorized for modeling, and non-linearly related to mortality. When complex sample design considerations were ignored, our method was generally similar to or more accurate than two common model selection procedures, Schwarz’s Bayesian Information Criterion (BIC) and Akaike’s Information Criterion (AIC), in terms of correctly selecting the correct number of knots. Our approach provided accurate knot selections when complex sampling weights were incorporated, while AIC and BIC were not effective under these conditions.

## Introduction

Large epidemiological surveys are powerful sources of observational information for investigating health outcomes as they relate to potentially predictive variables in the presence of confounding factors. The datasets from many of these surveys, such as the National Health and Nutrition Examination Survey (NHANES) and the National Health Initiative Survey (NHIS), are complicated by the fact that the surveyed participants are not selected by simple random sampling (SRS). The survey designers planned the sampling of groups of individuals in multiple stages with oversampling of certain demographic or geographic clusters to collect a complex sample which represents the population more efficiently than SRS.

There is a drawback to these designs in terms of additional statistical considerations that impact analyses of their data. Since equal probability of selection is not granted to each unit in the population and they are not independently sampled, observations taken from them should not be considered independent and identically distributed (iid). To illustrate why incorporating the sample design components, particularly the sample weights, is important in analyzing these data, consider this simple hypothetical example. Suppose that in the population you have 20% African Americans and 80% Caucasians and that due to planned oversampling, you have drawn a sample consisting of 50% African Americans and 50% Caucasians. Now suppose that the effect you are studying is more pronounced in Caucasians than African Americans. If you do not adjust for the additional weight given to the African Americans, you will misspecify the variability estimates and run the risk of either missing or spuriously detecting effects or differences because of the bias induced by over-representation of African Americans in the analysis. Thus, analyzing a complex sample with methods designed for SRS samples will produce incorrect variance estimates and possibly biased estimates of means and model parameters.

Common traditional statistical methods for modeling and hypothesis testing have been adapted to account for the imbalances and correlations induced by the survey sampling design (1, 2). Specialized software packages, such as SUDAAN or WestVar, have been designed for conducting many types of common statistical analyses on complex samples. The freely available R statistical computing software with the survey package, described in detail elsewhere (3), gives the ability to incorporate complex sample design features into R modeling function calls. This provides the analyst a tremendous amount of flexibility in the types of models, including various types of non-linear models that can be fitted while taking into consideration complex sample designs. However, software is not currently available for modeling complex sample data by certain specialized modeling techniques, such as free-knot splines that will be described in detail below. Some have pointed out the utility of free-knot spline models for effectively representing non-linear associations between continuous predictors and a binary outcome (4). Interestingly, they also describe how some free parameters in their models can be interpreted as thresholds for distinguishing groups with differing risk relationships. This implies that these modeling techniques can be used to identify thresholds that may have important biological or clinical significance. As such, free-knot spline modeling methodology could become very useful in providing an alternative to traditional quantitative epidemiological methods for characterizing non-linear risk relationships (i.e., parsing an important continuous predictor into local pieces having distinct relationships with outcome).

### Objective

We propose in this paper a free-knot spline framework for conducting piecewise linear logistic regression in complex multistage survey samples using B-splines and bootstrapping with a focus on likelihood function maximization for model computation. Piecewise linear representations of parameter estimates and odds ratios (OR) are output for expressing results in a familiar-looking format. A study of simulated mortality outcomes conditioned on measured body mass index (BMI, kg/m^2^) data from a real complex sample will demonstrate the performance of the procedure we have developed for specifying the optimal number of knots under various population and sampling conditions. BMI and mortality data provide a particularly apt example and area of application for our modeling framework since BMI is commonly recorded in complex samples, often categorized for modeling, and non-linearly related to mortality (5).

## Free-Knot Splines Logistic Regression Modeling Framework

### Modeling BMI as a predictor of outcome

A key assumption in fitting linear models or generalized linear models, as is the case here, with a continuous predictor is that there is an underlying quantitative relationship between predictor, BMI, and outcome, mortality odds. Furthermore, we assume that mortality odds can be represented well by some estimable function of BMI (i.e., a functional form). There are basically three different ways of treating BMI to characterize a flexible functional form between BMI and mortality odds: (1) categorizing BMI, (2) using a polynomial basis expansion of continuous BMI, or (3) using a spline basis expansion of continuous BMI.

Many investigators have applied contiguous categories of BMI set *a priori* by common standards (e.g., underweight: BMI < 18; normal: 18 ≤ BMI < 25; overweight: 25 ≤ BMI < 30; and obese: 30 ≥ BMI) or by some other arbitrary classification rules. The motivation for categorizing BMI is most likely convenience or convention. Categorization can facilitate examination of differences between groups and does not assume linearity or smoothness in the outcome relationship. However, categorization of BMI has significant disadvantages and limitations, including ignoring within-category BMI information resulting in decreased statistical power; insensitivity of trend tests to non-monotonic relationships; trend tests may indicate a trend, but cannot describe it; similar individuals within a BMI category are treated as though they have a uniformly constant mortality odds regardless of their actual BMI level; the results can depend heavily upon how the categories are chosen; and unfortunately, *a priori* classification boundaries of BMI are not likely to represent “true” partitions that would group individuals according to the underlying pattern of outcome likelihood within a BMI category.

Categorical analysis of BMI can be used to effectively compare results across studies and to provide a useful approach to exploring the extent to which any function fitted to the continuous BMI data adequately captures the apparent pattern in the functional relationship and suggest alternative functions when indicated. Following comparative or explorative categorical analyses, however, it is most often appropriate and important to return to a continuous BMI model for representing its relationship with the outcome in order to avoid bias (6).

Treating continuous BMI with polynomial predictor variables does not degrade data, tends to preserve power, and does not impose arbitrary categories or groupings. Curved U- or J-shaped relationships are commonly detected in BMI and mortality data modeled with polynomials of BMI. Modeling with polynomials can also have disadvantages and limitations, including: lack of flexibility possibly leading to biased estimation, particularly in the tails of the BMI distribution; and poorly parameterized models that smooth over any real partitions between BMI groups having different mortality risk relationships.

Splines can offer some of the best features of continuous and categorical modeling of BMI.

### Brief general background on non-linear modeling with splines

The literature regarding innovation in non-linear modeling and smoothing methods in recent decades has focused in several areas (with cited examples): penalized splines with fixed knots (P-splines) (7, 8); multivariate adaptive regression splines (MARS) (9); incorporating splines into logistic regression (4, 10) and survival analysis (11–15); Bayesian methods that utilize Reversible Jump Markov Chain Monte Carlo to fit penalized splines (16) and Bayesian Adaptive Regression Splines (BARS) (17); applying mixed models to smoothing (8, 18); generalized additive models (GAM) (19, 20); and free-knot splines (21, 22). Some researchers are also applying bootstrapping methods to spline estimation (4, 15, 23). Research in spline methodology continues to be popular as a resource for new methods, particularly those utilizing the increased computing power of today’s technology. Splines applied to modeling may become increasingly important for summarizing information and drawing inferences from data sources that are growing in number and complexity.

### What free-knot splines offer, some of their limitations, and why they are used

Specialized statistical modeling tools are called for in clinical and epidemiological settings for constructing useful models under circumstances of non-linearity, non-normality, and heteroscedasticity, which represent departures from GLM assumptions (2). One such modeling tool is the free-knot spline. A free-knot spline may be loosely described as a non-linear regression characterized by piecewise polynomials of order *m* joined at locations called knots where the adjoining segments typically agree at their (*m*−2)th derivative and both the number and locations of the knots are free parameters estimated along with other model parameters (24). Free-knot splines can be used to generate models that incorporate “local” flexibility over contiguous regions of BMI that are defined by the locations of the knots. In these models, spline parameters are estimated simultaneously along with any other model parameters, such as covariate parameters. As we describe below in Section “Interpretability of the knots can be biologically or clinically useful,” the knots themselves are parameters that can be used under certain circumstances to estimate partition boundaries (also called cut-points, knots, or thresholds) characterizing groups experiencing differing, non-uniform BMI-mortality odds relationships. The localized estimation properties of these models limit the influence of observations to particular regions of the fitted model (22) and can also be particularly helpful for better characterizing associations in the tails of the predictor and response joint distribution where: (1) small proportions of perhaps the most interesting observations exist and (2) polynomial models lack flexibility and are more susceptible to the impacts of outliers and high-leverage data points. A disadvantage of using free-knot splines is that they can be challenging to estimate and apply as statistical software packages typically will not fit them automatically.

Molinari et al. (15) give an excellent discussion of how to utilize and interpret free-knot splines with knots in the log hazard function where mortality risk, expressed as a hazard ratio, may vary greatly. In a similar fashion, Keith et al. (25) used free-knot splines to show how increased BMI is associated with a steady decrease in risk of severe or frequent headache in women with BMI below a threshold of approximately 20 (an estimated risk threshold parameter in the population) and a steady increase in risk above 20. A similar analysis stemming from categorized BMI would only describe an average risk of headache within each arbitrary classification (say above vs. below the detected threshold of BMI = 20), thus giving little information regarding the patterns of risk over the complete range of BMI.

Our non-linear framework utilizes piecewise linear free-knot splines to build an additive model of a dichotomous outcome as a non-linear function of a continuous predictor. The knots are estimated as free parameters along with other linear continuous or categorical covariate parameters. Estimating the optimal number and locations of the knots improves the approximating power of the model, but has been marked by computational intensity and numerical instability (26). Free-knot splines are very sensitive to local maxima in either the likelihood or residual sums of squares (SSE) surfaces. Free knots also tend to coalesce or overlap. The result has often poor computational performance, which has been described as the “lethargy property” of free-knot splines (27). Some successful efforts have been made to mitigate these challenges with the introduction of B-splines (24) and penalties for coalescent knots (22).

The optimization of even one non-linear relationship via a free-knot spline has proven to be a difficult task in large datasets. If the computational demands and numerical instability associated with free-knot splines may be overcome, the free-knot models may have great potential for optimal non-linear model fit to observed data in many dimensions. In our present approach, however, we have restricted our methodology to non-linearity between an outcome and only one independent variable while adjusting for covariates. This helps simplify our presentation and simulations by at least avoiding the computational demands and instability attributable to the “curse of dimensionality” (19, 28), which can be described as the problem of extremely rapid increases in data sparseness as the dimension of the non-linear multivariate space increases.

A key feature of the framework is that the splines may be represented algebraically and interpreted according to their piecewise polynomial segments, which gives the output from these models a familiar appearance to researchers accustomed to interpreting GLM results. This is an important aspect as the framework is intended to be accessible and attractive for use by epidemiologists and other quantitative researchers.

### Interpretability of the knots can be biologically or clinically useful

Effectively estimating both the numbers and locations of the knots tends to produce a simpler, low dimensional analytic function than fixing either the number or locations (or both) of the knots *a priori*. This is appealing from the perspective of parsimonious model fitting, but can also provide an interesting interpretation for the knots. Assuming that the true model has the same order and number of knots as that estimated, then the model may be considered parametric. Some have used this to their advantage by interpreting the knots in their free-knot spline models as cut-points in a risk relationship that define thresholds between groups with differential patterns of association with the outcome of interest (4, 15). We suggest that this may be inappropriate for cubic, perhaps even quadratic, free-knot splines as the ability to correctly specify the true model in simulation studies decreases greatly with increasing order. However, in situations where the aforementioned assumption holds sufficiently well, this interpretation of the knot parameters can yield compelling biological or clinical insights.

### Basis functions

A spline is constructed from basis functions. A basis function is an element of the basis for a function space. Each function in a given function space can be expressed as a linear combination of its basis functions. For example, the class of cubic polynomials with real-valued coefficients has a basis consisting of 1, *x*, *x*^2^, and *x*^3^. Every cubic function can be written as a linear combination of this basis (i.e., *a*1 + *bx* + *cx*^2^ + *dx*^3^). Basis function expansions must be explicitly specified in order to calculate free-knot splines. We considered two possible bases for our framework: the truncated power basis (8) and the B-spline basis (24).

#### Truncated power basis

The truncated power basis expansion of order *m* can be expressed as

(1) $$f\left(x\right)=\beta_{0}+\beta_{1}x^{1}+\cdots +\beta_{p}x^{p}+\sum_{i=1}^{K}b_{\mathrm{pi}\;}{\left(x-\zeta_{i}\right)}_{+}^{p}$$

where some function, *f*, is a non-linear, piecewise polynomial (having degree = *p*) function of an independent variable, *x*; ζ*_i_* is the *i*th of *K* knots such that ζ_1_ ≤ ζ_2_ ≤ ⋯  ≤ ζ*_K_*; and *u*_+_ = max(*u*,0). Here, we limit our scope to the piecewise linear truncated power basis expansion (order *m* = *p* + 1 = 2)

(2) $$f\left(x\right)=\beta_{0}+\beta_{1}x+\sum_{i=1}^{K}b_{i}{\left(x-\zeta_{i}\right)}_{+}.$$

#### A piecewise linear representation

We use indicator functions, I{ }, to then express the truncated power basis as a piecewise linear function on the *i*th contiguous interval of the domain of the predictor vector variable, **X**, delimited by either the knots or bounds of **X**. Suppose that each numerical item, *x*, in this vector has the property *x* ∈ ℜ^+^ and that we fix knots that will not be estimated at the endpoints *a* = min(**X**) and *b* = max(**X**) such that ζ_0_ = *a* and ζ*_K_*_+1_ = *b*, then we have

(3) $$\begin{array}{llll} h\left(x\right) & =a_{0}+a_{1}x\;I\left\{x<\zeta_{1}\right\}+\sum_{i=1}^{k}\left[a_{i+1}\left(x-\zeta_{i}\right)\right.\; & & \;\\ & \left.\;+\sum_{j=1}^{i}a_{j\;}\left(\zeta_{j}-\zeta_{j-1}\right)\right]I\left\{\zeta_{i}\leq x<\zeta_{i+1}\right\} \end{array}$$

where *a*_1_ = β_1_ using the coefficients from (2) can give us

(4) $$a_{l}=a_{1}+\sum_{i=1}^{l-1}b_{i},\;\left(l=2,\;\ldots ,\;K+1\right)$$

the slope parameter for any observed *x* ∈ [ζ*_l_*_−1_, ζ*_l_*]. Note that this basic transformation is used only for estimating the slopes after optimization. Although algebraically equivalent, this basis representation of the piecewise linear space is less computationally stable for optimization than the truncated power basis.

To illustrate our model including covariates, suppose that **X** is an *N* Œ 1 vector of data on some continuous prognostic variable of interest and **Z** represents a *N* Œ (*p* + 1) matrix consisting of a column of ones followed by *p* columns of data on covariates. Let η be a parametric function of *p* + 1 linear covariate predictors multiplied by their respective logistic regression coefficients (***β***) and the *K* + 1 piecewise linear slope coefficients (*a*_1_,…, *a_K_*_+1_). For the *q*th individual (*q* = 1, …, *N*)

(5) $$\begin{array}{llll} \eta \left(\left[{\text{Z}}_{q},X_{q}\right]\right) & =\beta_{0}\;+\;\beta_{1}Z_{q2}+\cdots +\beta_{p}Z_{q\left(p+1\right)}\;+\;a_{1}X_{q}I\left\{X_{q}\;<\;\zeta_{1}\right\}\; & & \;\\ & \;+\sum_{i=1}^{K}\;\left[a_{i+1}\left(X_{q}-\zeta_{i}\right)+\sum_{j=1}^{i}a_{j\;}\left(\zeta_{j}-\zeta_{j-1}\right)\right]\; & & \;\\ & \;\times I\left\{\zeta_{i}\leq x<\zeta_{j-1}\right\}. \end{array}$$

For comparison, consider the simpler truncated power basis expression

(6) $$\begin{array}{llll} \eta \left(\left[{\text{Z}}_{q},\;X_{q}\right]\right) & =\beta_{0}+\beta_{1}Z_{q2}+\cdots +\beta_{p}Z_{q\left(p+1\right)}+b_{0}X_{q}\; & & \;\\ & \;+{\sum_{i=1}^{k}b_{i}\left(X_{q}-\zeta_{i}\right)}_{+}. \end{array}$$

#### B-splines

B-spline bases are easy to incorporate into the framework by applying de Boor’s recurrence relation for their practical implementation (24). B-splines are used extensively throughout the non-linear modeling literature. Here, we discuss them only in brief detail.

Consider a knot sequence, ζ_0_ = min(**X**) = ζ_1_ ≤ ζ_2_ ≤ ⋯  ≤ ζ*_K_*  ≤ ζ*_K_*_+1_ ≤ ζ*_K_*_+2_ = max(**X**) = ζ*_K_*_+3_, such that there are *K* interior knots {ζ_2_, …, ζ*_K_*_+1_}. By the definition of B-splines, the *j*th B-spline of order *m* = 1 (piecewise constant) is

(7) $$B_{j1}=\left\{\begin{array}{cc} 1\; & \mathrm{if}\;\zeta_{j}\leq X_{q}<\zeta_{j+1}\\ 0\; & \mathrm{otherwise}\\ \; \end{array}\right.$$

and the higher order B-splines may be constructed by this recurrence relation

(8) $$B_{\mathrm{jm}}=\omega_{\mathrm{jm}}{B_{j}}_{\left(m-1\right)}+\left(1-\omega_{\left(j+1\right)m}\right)B_{\left(j+1\right)\left(m-1\right),}$$

where

(9) $$\omega_{\mathrm{jm}}\left(X_{q}\right)=\frac{X_{q}-\zeta_{j}}{\zeta_{j+\text{m}-1}-\zeta_{j}}.$$

So the linear B-spline basis of order *m* = 2 (piecewise linear) we use can be expressed for any *X_q_* ∈ ℜ as

(10) $$\begin{array}{llll} \beta_{j2} & =\frac{x_{q}-\zeta_{j}}{\zeta_{j+1}-\zeta_{j}}I\left[\zeta_{j}\leq X_{q}<\zeta_{j+1}\right]+\frac{\zeta_{j+2}-X_{q}}{\zeta_{j+2}-\zeta_{j+1}}\; & & \;\\ & \;\times I\left\{\zeta_{j+1}\leq X_{q}<\zeta_{j+2}\right\},\;j=0,\;\ldots ,\;K+1, \end{array}$$

where *K* is the number of interior knots fitted. Thus we have

(11) $$\begin{array}{llll} \eta \left(\left[{\text{Z}}_{q},X_{q}\right]\right) & =\text{Z}q\beta +{\text{B}}_{q}\text{b}=\beta_{0}+\beta_{1}{Z_{q}}_{2}+\cdots +\beta_{p}Z_{q\left(p+1\right)}\; & & \;\\ & \;+\sum_{i=1}^{K+1}b_{i}B_{qi2}\left(X_{q}\right), \end{array}$$

where *b*_1_, …, *b_K_*_+1_ are linear regression coefficients corresponding to their respectively indexed values in the *q*th row vector, **B***_q_*, of the B-spline expansion matrix **B**. This shows how η is an additive, linear expression of B-spline parameters. Note that Eq. 11 may be easily transformed to a polynomial expression as piecewise polynomial coefficients are clearly linear combinations of the B-spline coefficients.

### Computation methods

Before we discuss optimizing the fit of the spline to data, we briefly consider some computational aspects. Mathematicians and computer scientists have demonstrated that B-splines can have desirable properties, such as local linear independence (24) and computational stability (29). As such, B-splines have been a popular choice used extensively for free-knot modeling (4, 22).

We are fitting non-linear functions constrained to the class of piecewise linear free-knot spline functions mapping a continuous independent variable onto the space of the outcome variable as a projected estimate of the mean response surface. Our goal is to first find the optimal fit for a given number of knots, *K*, and then determine which value of *K* best represents the data. There are two general approaches to these computations:

(1) by minimizing the sum of squared distances between observed and predicted values (i.e., leastsquares estimation or LSE) and
(2) by maximizing the likelihood function (i.e., maximum likelihood or MLE approach).

#### Least squares

LSE in this context involves minimizing, with respect to residual SSE, the distance between the observed outcome or a function of the observed outcome and non-linear estimates. This typically requires a method, such as the Gauss–Newton method with the Levenberg–Marquardt adjustment (30, 31), which uses derivatives or estimates of derivatives to pick out the optimal fit.

No canned SAS procedures (SAS Institute, Cary, NC, USA) such as PROC GAM, PROC TRANSREG, or PROC TPSPLINE are capable of fitting free-knot splines. However, a free-knot spline basis can be computed at run time with SAS macros that use PROC NLIN for least-squares model estimation. This involves minimizing a measure of distance between vectors, say ${∥\text{f}-\hat{\text{f}}∥}^{2},$ which represents the non-linear SSE in a multidimensional space where **f** is the collected data and $\hat{\text{f}}$ is a collection of non-linear estimates as a function of the data, complex sample weights, and model parameters including free-knots.

#### Maximum likelihood

For MLE, the non-linear logistic likelihood function must be numerically maximized to find the parameter values under which the observed data were most likely produced. In theory, these estimates might have the properties of asymptotic efficiency and invariance under reparameterization which makes MLE attractive in general (32). This invariance property is particularly important to our framework as we intend to perform the optimization with B-splines and then reparameterize the estimates as linear combinations of B-spline parameters that will represent the local piecewise linear slopes. In practice, unlike the B-spline parameters, these slopes are straightforward and easy to present and interpret.

The Nelder–Mead simplex (33) is a popular and powerful direct search procedure for likelihood-based optimization. The attraction of this method is that the simplex does not use any derivatives and does not assume that the objective function being optimized has continuous derivatives. Nelder–Mead simplex optimization is the only method currently available in SAS, which does not require derivative calculations to search the parameter space. In cases such as ours (i.e., piecewise linear splines), we do not expect continuity in the first derivatives at the knot locations. Therefore an MLE and simplex optimization approach seems more reasonable than the LSE and residuals SSE minimization approach. Direct search methods can, however, be much less efficient or highly unstable as compared to derivative-based LSE or MLE methods when sample sizes are as large as the datasets common to complex survey designs. Hence, as a compromise, we have used “quasi-Newton methods” with estimated derivatives to perform the MLE.

##### Non-linear logistic likelihood

Let us now examine the non-linear logistic likelihood function for modeling binary outcomes. The probability of the *q*th participant having experienced the outcome of interest, *Y_q_* = 1, can be expressed as

(12) $$\begin{array}{llll} \pi_{q}\left(\eta \left(\left[{\text{Z}}_{q},\;X_{q}\right]\right)\right) & =P\left(Y_{q}=1|\eta \left(\left[{\text{Z}}_{q},\;X_{q}\right]\right)\right)\; & & \;\\ & =\frac{\exp\left\{\eta \left(\left[{\text{Z}}_{q},\;X_{q}\right]\right)\right\}}{1+\exp\left\{\eta \left(\left[{\text{Z}}_{q},\;X_{q}\right]\right)\right\}}, \end{array}$$

where η([**Z***_q_*, *X_q_*]) may be Eq. 11. Note that the *logit* or *log*(odds) function of this probability

(13) $$\begin{array}{llll} \mathrm{logit}\left\{\pi_{q}\left(\eta \left(\left[{\text{Z}}_{q},\;X_{q}\right]\right)\right)\right\} & =\log\left\{\frac{\pi_{q}\left(\eta \left(\left[{\text{Z}}_{q},\;X_{q}\right]\right)\right)}{1-\pi_{q}\left(\eta \left(\left[{\text{Z}}_{q},\;X_{q}\right]\right)\right)}\right\}\; & & \;\\ & =\eta \left(\left[{\text{Z}}_{q},\;X_{q}\right]\right), \end{array}$$

may reasonably be modeled piecewise linearly as a function of the variables in [**Z*_q_***, X*_q_*]. We may express a weighted likelihood function:

(14) $$L\left(\theta |\left[\text{Z},\text{X}\right],\text{W}\right)=\prod_{q=1}^{n}{\left[\pi_{q}{\;}^{y_{q}}{\left(1-\pi_{q}\right)}^{1-y_{q}}\right]}^{wq},$$

where ***θ*** is a vector of all the linear and spline parameters expressed in Eq. 11, *n* = sample size, π*_q_* is defined above in Eq. 12, *y_q_* is the binary outcome, and *w_q_* is the complex sample weight in the weight vector, **W**, assigned to the *q*th participant by the study designers. The weighted log-likelihood, which is more convenient for use in optimization procedures,

(15) $$\log\left\{L\left(\theta |\left[\text{Z},\text{X}\right],\text{W}\right)\right\}=\overset{n}{\underset{q=1}{\sum }}w_{q}\left[\log\left(1\;-\;\pi_{q}\right)+y_{q}\log\left(\frac{\pi_{q}}{1\;-\;\pi_{q}}\right)\right]\;.$$

may be maximized numerically using PROC NLP in SAS.

#### Optimization

Our goal is to first find the optimal fit for a given number of knots and leave the optimization with respect to the number of knots for the next section.

##### Quasi-Newton methods for MLE

In our experiences with tuning the optimization algorithm for analysis of real and simulated data, using quasi-Newton methods has produced more efficient and more stable results than the Nelder–Mead simplex. Quasi-Newton methods are a class of optimization algorithms which we used to locate minima in the negative natural logarithm of Eq. 14. The particular quasi-Newton procedure we employed is called the dual Broyden–Fletcher–Goldfarb–Shanno method (DBFGS) (34–37). The details of this procedure extend beyond the scope of this paper. In brief, DBFGS uses line searches along feasible descent search directions in combination with estimation of the Cholesky factor of the Hessian matrix of second derivatives to iteratively update the overall search for minima. Although this method requires first derivatives, we were able to calculate derivative estimates by using finite difference approximations as we did for the LSE methods. As expected, in application to large survey datasets, we have found that the MLE methods suffer fewer problems with convergence than the LSE methods. The non-linear LSE optimization procedure by the Gauss–Newton method is fairly straightforward, though, and additional information on this procedure is provided in Supplemental Material (see Quasi-Newton Methods for MLE).

#### Computing odds ratios

We found OR to be a powerful way of expressing event risk as a function of the non-linear predictor. We choose OR over the log(odds) when models have been adjusted for covariate information because, unlike log(odds), OR for comparing two otherwise similar individuals do not depend on the covariates. While computing OR in our framework is not quite as simple as in a conventional GLM, it is straightforward. Assume the basis in Eq. 5 and, assuming all else is equal between individuals *l* = 1 and *l* = 2 except for their respective non-linear predictor values, *X*_1_ and *X*_2_, respectively, we may compute an odds ratio:

(16) $$\mathrm{OR}=\frac{\begin{array}{c} a_{1}X_{1}I\left\{X_{1}<\zeta_{1}\right\}+\sum_{i=1}^{K}\left[a_{i+1}\left(X_{1}-\zeta_{i}\right)\right.\\ \left.\;+\sum_{j=1}^{i}a_{j}\left(\zeta_{i}-\zeta_{j-1}\right)\right]I\left\{\zeta_{i}\leq X_{1}<\zeta_{i+1}\right\} \end{array}}{\begin{array}{c} a_{1}X_{2}I\left\{X_{2}<\zeta_{1}\right\}+\sum_{i=1}^{K}\left[a_{i+1}\left(X_{2}-\zeta_{i}\right)\right.\\ \left.\;+\sum_{j=1}^{i}a_{j}\left(\zeta_{i}-\zeta_{j-1}\right)\right]I\left\{\zeta_{i}\leq X_{2}<\zeta_{i+1}\right\} \end{array}}.$$

Graphical representations of this OR may be created if a suitable reference level can be fixed for *X*_2_ while allowing *X*_1_ to range.

### Knot selection

#### A novel parametric bootstrap-based method

We outline in this section a novel method of selecting the optimal number of knots. Knot locations, linear and non-linear coefficients, and a common intercept are parameters optimized simultaneously while having complex sample weights incorporated into the fitted function. This achieves adjusted and, presumably, unbiased parameter estimates. Like others (4, 15), we are interested in interpreting the fitted knots to define *clinically meaningful* groups with differential patterns of risk. It is very important to correctly specify a parsimonious number of knots, say 4 or fewer, which would indicate 5 or fewer different risk groups. Therefore, keeping the framework from producing models with unnecessary knots is a priority.

Our technique involves a forward selection procedure based on the concept of a 2 df for the addition of two parameters, a knot and a slope, to the piecewise linear model (our “2 df knot testing procedure”). As depicted in Eq. 11, we are considering a set of *p* linear or categorical covariates for adjustment purposes, but this procedure is targeted at optimizing the complexity necessary to effectively model the one potentially non-linear prognostic variable, *X*. The test statistic for the LSE framework is an *F*-ratio:

(17) F=(SSEreduced−SSEfull)/2SSEfulldffull,

where the df of the full model, df_full_, is *N* – (*p* + 2*K* + 2) [i.e., the sample size minus the number of free parameters estimated: *p,* linear coefficients, *K,* free-knots, the *K* + 1, spline parameters (the piecewise linear slopes), and the intercept]. We are not certain of the distribution of *F*, so we use parametric bootstrapping (38, 39) to build a hypothetic distribution of these *F*-ratio test statistics under the null hypothesis that the reduced model having *K* knots is true against an alternative having *K* + 1 knots. We draw *D*_1_ parametrically resampled replicate datasets of binary outcomes and compute the *F*-ratio distribution $\left\{F_{1}^{\mathrm{rep}},\;\ldots ,\;F_{D1}^{\mathrm{rep}}\right\}.$ A bootstrap *p*-value representing the probability that adding the (*K* + 1)^th^ knot produces an *F*-ratio at least as large as what might be observed by chance alone can be calculated from this *F*-ratio distribution as

(18) $$p_{\mathrm{boot}}=\frac{1+\sum_{j=1}^{D_{1}}I\left\{F_{j}^{\mathrm{rep}}\geq F\right\}}{D_{1}+1}.$$

This is analogous to integrating the distribution of *F* to determine the probability of observing the data given that the null model is true. For the MLE, we adopted a similar approach, but with likelihood ratio (LR) test statistics:

(19) LR=−ln Lθ^null−ln Lθ^alt.

in place of the *F*-ratio statistics for comparing the minimized negative-log-likelihood functions from two models: a null model having *K* knots and parameter estimates for their locations and other regression estimates for parameters expressed in Eq. 11, ${\hat{\theta }}_{\mathrm{null}},$ versus an alternative model with a parameter estimate set, ${\hat{\theta }}_{\mathrm{alt}.},$ expanded from the null model to include an additional knot parameter and slope parameter.

We select a value for α to represent the significance level selection criterion for this test of the contribution to reducing SSE or increasing the likelihood. That is, the bootstrap *p*-value in Eq. 18 would have to be smaller than α in order to reject the null hypothesis that the model with *K* knots is the true model in favor of the model with *K* + 1 knots. We can control the flexibility of the model by manipulating α.

##### The algorithms

The details and algorithms for applying the LSE and MLE approaches to the 2 df knot testing procedures are shown in Tables S1 and S2 in Supplementary Material. By either approach, we end with output parameters, ${\hat{\theta }}^{\text{PLS}}$, for an optimal piecewise linear model expressed in terms of local linear slope coefficients, knot locations, and covariate coefficients.

#### Grid search

The selection of good starting values is critical for iterative optimization procedures in avoiding locally optimal model parameter settings in favor of converging to the global optima. It can be challenging to identify such starting values when modeling with free-knot splines. This issue is ubiquitous in the literature and is particularly troublesome in regions where the functional surface relating the non-linear predictor and response is nearly flat. Not only is it important to place the knots well, but the algorithm must also start with well-placed covariate and spline parameters. To address this, we start spline coefficient parameters at zero and any covariate coefficient parameters at their multivariate GLM estimates. For the knots, we objectively search the free-knot parameter space for plausible knot locations by using a grid search algorithm similar to that applied by others (4). This obviates the need for subjectivity in assigning starting values, but comes with high computational costs as increasing the size of the grid has a multiplicative impact on the number of times we need to run the bootstrap testing procedure.

The grid search was implemented in steps 4 and 5 of the 2 df MLE testing procedure algorithm to locate the best starting values, $\theta_{\text{null}}^{0}$ and $\theta_{\text{alt.}}^{0}$ (see Table S2 in Supplementary Material). That is, we set starting covariate coefficient parameters in ***θ***^0^ equal to those estimated by a linear logistic regression in SAS Proc Survey-Logistic. For the MLE, we calculate models for *C* possible starting locations placed at nearly equal distances throughout the range of the predictor to avoid getting stuck on local maxima and help prevent coalescent knots. To ensure that knots did not overlap, we also enforced linear constraints so that a small minimal distance was maintained between any two knots, including the boundary knots. As noted, the search can be extremely computationally expensive as for each loop from step 2 to 9 of the algorithm, we must make ${(}_{K}^{C})$ calls to fit a model with PROC NLP. In the most extensive case we consider, where we reject the model having *K* = 2, we would require a total of $4+(D_{1}+1)\times \{{(}_{0}^{C})+2\times [{(}_{1}^{C})+{(}_{1}^{C})+{(}_{3}^{C})]+{(}_{4}^{C})\}$ PROC NLP calls, where D_1_ represents resampled replicate datasets. For instance, this quantity might range from 22,604 if *D*_1_ = 200 and *C* = 6 up to 511,004 if *D*_1_ = 1,000 and *C* = 9. The latter represents a large number of program calls and is recommended only for high performance parallel processor computing environments.

### Estimating uncertainty in parameter estimates

#### Incorporating multistage probability cluster sampling

The data we are considering are drawn from the target population using complex, multistage probability cluster sampling that achieves the quality of effectively representing the population much more quickly than the classic SRS design (1, 40). There are three components to the information provided to the analyst to adjust for the unequal probability sampling of multistage complex sample designs we see in datasets such as NHANES and NHIS. The components are stratum, primary sampling unit (PSU), and sample weight. The strata are often based on geographic area. PSUs are clusters within a stratum and generally given a probability of being selected for sampling that is proportional to the size of the cluster (with the exception that some clusters, such as the New York City metropolitan PSU in NHANES that are assigned a selection probability = 1). The sample weights can be loosely defined as giving each sampled participant a weight to indicate what proportion of the population they represent (i.e., what proportion of the population has the same apparent characteristics as a given sampled participant).

The complex sample design variables actually presented to the interested researcher are pseudo-variables. They have been modified by the survey designers in order to protect participant confidentiality by masking the true sampling design features, but maintain their useful utility for providing unbiased parameter estimates and standard errors. It is not clear from the pseudo-variables which PSUs have been sampled with certainty and which have not.

#### Making adjustments without existing software

As we are not aware of any available tools, such as SUDAAN software or R with the survey package, for free-knot spline modeling of survey data with complex sample designs, we wrote our own *ad hoc* software utilizing available methodology to make appropriate adjustments in our programs. There are two basic approaches to making complex sample adjustments: linearization and resampling. Linearization is the application of a Taylor’s series expansion to make first order linear approximations to possibly non-linear parameters. Variance estimates are then based on the linear approximations (41). Some have provided useful ideas for alternative approaches to this problem based on resampling (42). Rao (41) suggests that

“An advantage of a resampling method is that it employs a single standard-error formula for all statistics, $\hat{\theta },$ unlike the linearization method, which requires the derivation of a separate formula for each statistic $\hat{\theta }.$ Moreover, linearization can become cumbersome in handling poststratification and non-response adjustments, whereas it is relatively straightforward with resampling methods… As a result, they [software packages using linearization] cannot handle more complex analyses such as logistic regression with poststratified weights.”

Thus, resampling provides a more general and versatile approach well suited to our problem.

The resampling methods detailed by Rao et al. (42) include balanced repeated replication (BRR), the jackknife, and bootstrap. BRR involves resampling many “half-sample” replicates by deleting one PSU from each stratum, rescaling the complex sample weights, calculating a weighted replicate parameter estimate, and computing variance estimates for the original parameter estimate based on the variability in the BRR replicates. This method does not work well in cases where we have more than two PSU per stratum. The jackknife method deletes one PSU, rescales the sample weights, calculates a replicate parameter estimate, and repeats this for each PSU within each stratum. A variance estimate for the original parameter estimate can then be calculated from these jackknife replicates.

The most convenient resampling approach is to resample the PSUs with replacement within each strata by using the non-parametric bootstrap method (42) and appropriately rescale the weights. To be specific, the individual sampling weights within the *h*th stratum (*h* = 1, …, *H*) are rescaled by the following equation:

(20) $$w_{\mathrm{hij}}^{*}=w_{\mathrm{hij}}\left(1-\sqrt{\frac{d_{h}}{n_{h}-1}}+\sqrt{\frac{d_{h}}{n_{h}-1}\times \frac{{\text{n}}_{h}}{d_{h}}\times r_{\mathrm{hi}}}\right).$$

where $w_{hij}^{*}$ is the rescaled weight for *j*th individual in the *i*th PSU, *w_hij_* is the original weight for the *j*th individual in the *i*th PSU, n_*h*_ and *d_h_* are, respectively, the number of PSUs and the number of bootstrap samples drawn from this stratum, and *r_hi_* is the number of times the *i*th PSU is resampled. This is the underlying methodology applied in our framework to achieve approximately unbiased standard errors and confidence intervals adjusted for multistage complex sample designs.

Some have presented detailed discussions of this method for bootstrap adjustment of complex multistage sample weights when the number of PSUs per statum is at least 2 (n_*h*_ ≥ 2) (42, 43). Rao et al. (42) suggested that this method is valid and consistent for estimated parameters expressed as either smooth or non-smooth functions of totals when *n*_h_ ≥ 2 and *H* is relatively large (e.g., *H* = 49 in NHANES III). Setting *n_h_* = 2 is a popular choice (common to both the NHANES and NHIS series) as it provides the maximum amount of stratification possible for conducting valid variance estimation.

Once we have settled on a model with *K* knots by application of our 2 df knot testing procedure, we are prepared to ascertain the certainty in our parameter estimates. The specification for our complex sample adjustment procedure is outlined in Table S3 (corresponding to the LSE approach) and Table S4 (corresponding to the MLE approach) in Supplementary Material. We begin by applying the methods suggested by Rao et al. (42) described above to generate D_2_ non-parametric bootstrap replicate estimates per each parameter of interest. Investigators have applied the LSE methodology to BMI as a predictor of binary headache outcomes in women (25), used the bootstrap-*t* method described by DiCiccio and Efron (44) for calculating 95% CI from *D*_2_ = 1000 non-parametric bootstrap replicates. Some have suggested this method as a general guideline for improving statistical power and the accuracy of coverage probabilities [i.e., bootstrapping a distribution for an asymptotically pivotal quantity, $T=\left(\hat{\theta }-\theta \right)∕\hat{\sigma }$ by $T_{i}^{*}=\left({\hat{\theta }}_{i}^{*}-\hat{\theta }\right)∕{\hat{\sigma }}_{i}^{*},\;i=1,\ldots ,D_{2},$ where θ is some parameter of interest (say a particular knot or slope), $\hat{\theta }$ is the original parameter estimate, $\hat{\sigma }$ is the original standard deviation estimate, ${\hat{\theta }}_{i}^{*}$ and ${\hat{\sigma }}_{i}^{*}$ are the parameter and standard deviation estimates, respectively, from the *i*th bootstrapped sample] (45). Then the bootstrap estimate of the standard error of $\hat{\theta }$ is

(21) $${\hat{\sigma }}^{*}=\sqrt{\frac{1}{D_{2}-1}{\left({\hat{\theta }}^{*}-{\bar{\hat{\theta }}}^{*}\right)}^{T}\left({\hat{\theta }}^{*}-{\bar{\hat{\theta }}}^{*}\right)}$$

where ${\hat{\theta }}^{*}$ represents the vector of ${\hat{\theta }}_{i}^{*}$’s estimated from the D_2_ bootstrap samples and ${\bar{\hat{\theta }}}^{*}=\left(1∕D_{2}\right)1^{T}{\hat{\theta }}^{*}$ is the mean of the bootstrap replicates.

The distribution of *T* is not necessarily symmetric, so we locate the critical values at either end of the ordered bootstrapped distribution ${\text{T}}^{*}=\left\{T_{\left(1\right)}^{*},\ldots ,T_{\left(D_{2}\right)}^{*}\right\}$ such that *P*(T*_(lower critical)_ < *T*  < T*_(upper critical)_) ≥ 0.95, with equal probability in either tail, and applying some algebra leads to the 95% CI for $\theta =\left({\hat{\theta }}_{\left(\mathrm{lowercritical}\right)}^{*},\;{\hat{\theta }}_{\left(\mathrm{uppercritical}\right)}^{*}\right)$, where ${\hat{\theta }}_{\left(\mathrm{lowercritical}\right)}^{*}=T_{\left(\mathrm{lowercritical}\right)}^{*}{\hat{\sigma }}_{\left(\mathrm{lowercritical}\right)}^{*}-\hat{\theta }$, and ${\hat{\theta }}_{\left(\mathrm{uppercritical}\right)}^{*}=T_{\left(\mathrm{uppercritical}\right)}^{*}{\hat{\sigma }}_{\left(\mathrm{uppercritical}\right)}^{*}-\hat{\theta }$.

This method can be more stable and less conservative than using the more basic percentile methods (39) and applied to free-knot splines (4), however, the standard-error estimates ${\hat{\sigma }}_{i}^{*}$ were drawn from the optimization procedure (PROC NLIN) and required running the model with the far less stable piecewise linear basis, depicted in Eq. 5, in order to apply them directly to the bootstrap-*t* distribution of the slope coefficient parameters.

### Simulations: Evaluating the MLE 2 degree of freedom knot testing procedure

A simulation study was devised to assess how well the MLE 2 df knot testing procedure performs in correctly specifying the optimal number of knots and compare results to those obtained by other popular model selection criteria:

(22) $$\mathrm{AIC}=-2\;\log\left\{L\left(\hat{\theta }|X,\;W\right)\right\}+2r$$

and

(23) $$\mathrm{BIC}=-2\;\log\left\{L\left(\hat{\theta }|X,\;W\right)\right\}+r\log\left(n\right),$$

where *r* represents the number of parameters in the model and n is the sample size. The conditions we focused upon in simulating data were the size of the sample (*n*) and the proportion of events (*p*_o_). For each, we selected two setting: *n* ∈ {500, 5000} and *p_o_* ∈ {0.10, 0.33}. The simulated outcomes data were plasmodes (46) generated from randomly selected samples of *n* BMI records from NHANES III – a complex sample weighted, nationally representative survey conducted between 1988 and 1994 with mortality follow-up in 2000. Plasmodes are sets of data generated by natural biologic processes (i.e., hypothetical outcomes data generated conditional on real BMI from a real complex sample) under experimental conditions we set. This allows us to evaluate an aspect of truth (i.e., the rate at which models select the correct number of knots under these conditions) in a realistic simulated environment. One hundred simulated sets of n binary mortality outcomes were generated conditional on the *n* BMI records and a true *log*(odds) model having two knots in their piecewise linear relationship. More precisely, for a given set of n BMI values, each one, *q* = 1, …, *n*, was assigned a Bernoulli random variable, *Y_qj_* | *X_q_* = BMI*_q_* (*j* = 1, …, 100), based on the probability of event defined by

(24) $$\pi_{q}\left(\eta \left(\left[X_{q}\right]\right)\right)=P\left(Y_{q}=1|\eta \left(\left[X_{q}\right]\right)\right)=\frac{\exp\left\{\eta \left(\left[X_{q}\right]\right)\right\}}{1+\exp\left\{\eta \left(\left[X_{q}\right]\right)\right\}},$$

where the η function was specified by a true log(odds) model having *K*_true_ = 2 knots fixed at BMI = 25 and BMI = 32 and piecewise slopes fixed at *a*_1_ = −0.4, *a*_2_ = 0.0, and *a*_3_ = 0.2 as in (3). These parameter settings were chosen as they define a functional shape that characterizes the true non-linear U-shaped BMI relationship with a binary mortality outcome variable observed during follow-up among NHANES III participants having 17 ≤ BMI ≤ 45 at their baseline assessment. The intercept of the true model, *a*_0_, was calculated for each BMI dataset as it must be conditioned on the desired level of *p*_o_. To evaluate sensitivity to the selection criterion (α), we ran our MLE 2 df knot testing procedure with settings of α ∈ {0.10, 0.25} on all combinations of the *n* and *p*_o_ settings.

**Figure 1:**
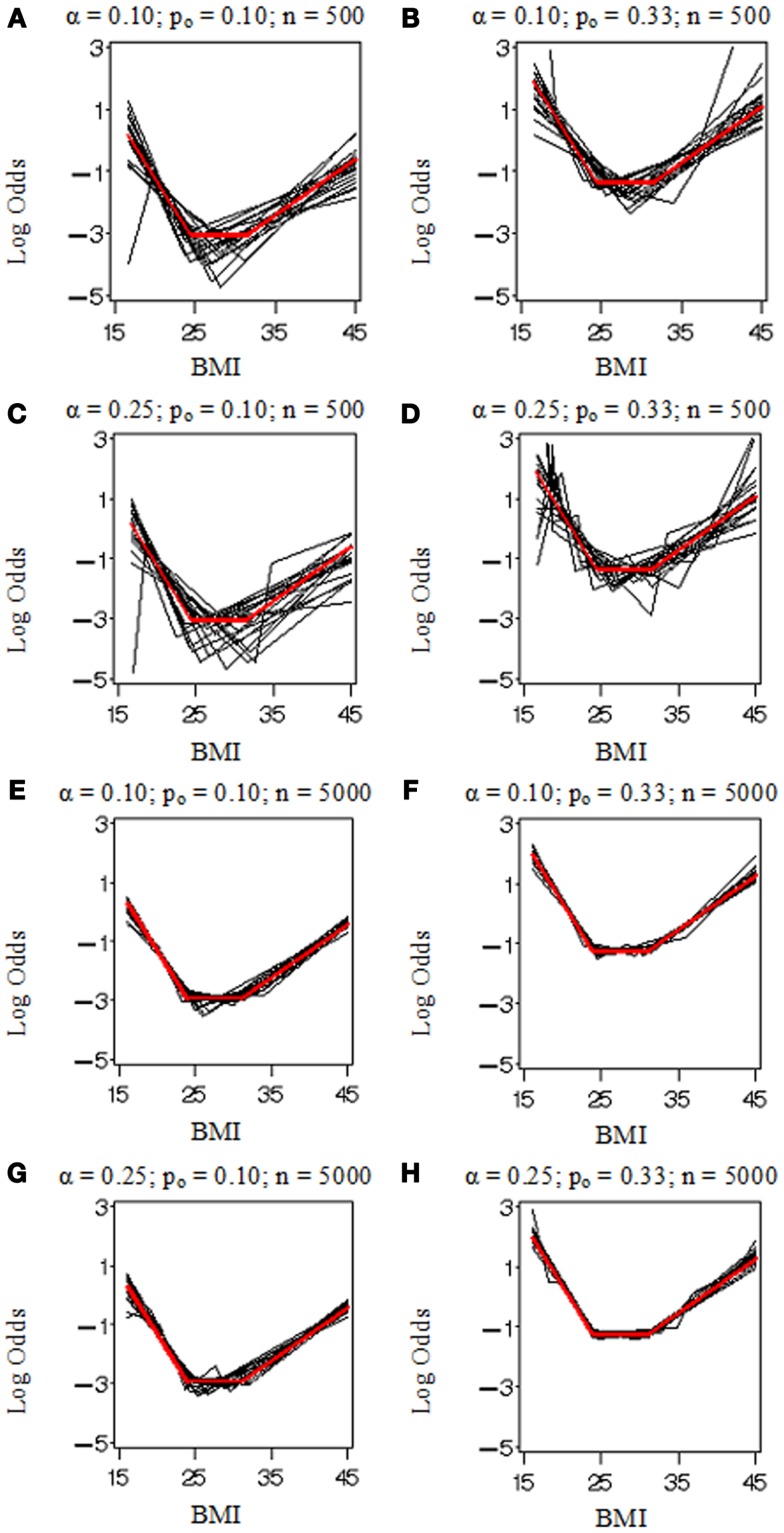
**Model selection simulation results for the parametric bootstrap MLE 2 df selection procedure**. True log odds model (*K*_true_ = 2) plotted (in red) by BMI with results from 20 replicate datasets (in black). Each **(A–H)** depicts results from data simulated under various conditions including the proportion of events, *p*_o_ (0.10 or 0.33) and sample size, *n* (500 or 5,000), and evaluated by our methods with two selection criterion settings, α (0.10 or 0.25).

**Figure 2:**
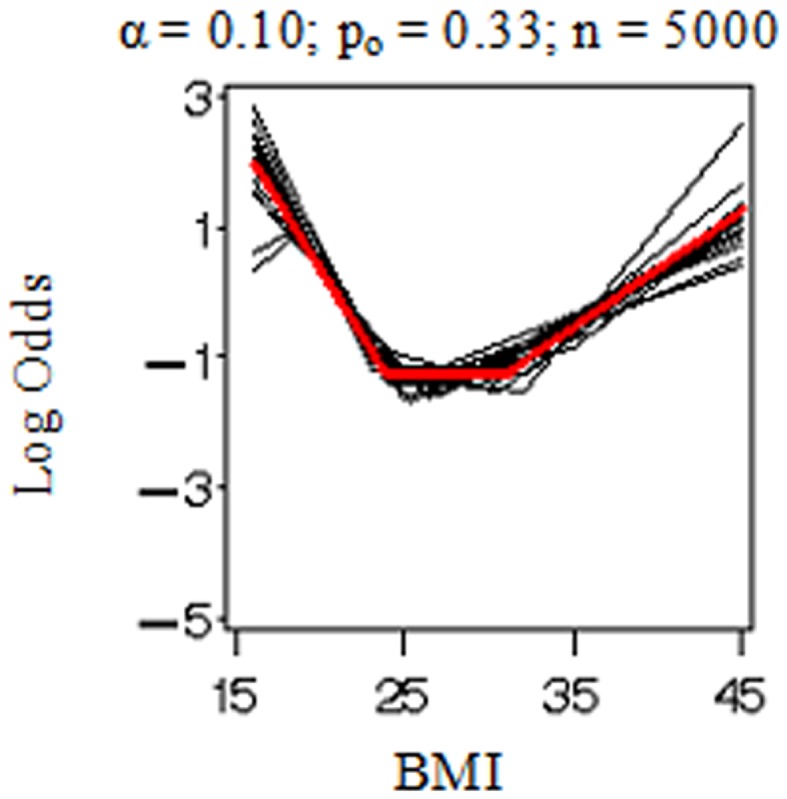
**Model selection simulation results when sampling weights are applied**.

Figure 1 displays the true model (in red) and replicated models (in black) resulting from the application of the MLE 2 df knot testing procedure to 20 randomly selected simulated datasets for each combination of settings. Note that if the model having *K* = 3 was rejected, we selected the model with *K* = 4 and stopped the evaluation at that point in order to conserve computation time. When the sample size was low (*n* = 500 in Figures 1A–D), there was considerable error variance or “noise” distorting the true model “signal” which generated the binary simulated data and our procedure did not perform nearly as well as when there was more information available (*n* = 5,000 in Figures 1E–H). When the proportion of events was elevated (*p*_o_ = 0.33 in Figures 1B,D,F,H), it also introduced more information and reduced uncertainty in where the true model was located. Figure 2 shows an instance in which the sample weights from NHANES III had been included. Incorporating the sample weights did not appear to greatly impact the accuracy of the MLE 2 df knot testing procedure, but it did introduce an extra source of variance and possibly some degree of numerical instability resulting in increased computation time and lower precision.

**Figure 3:**
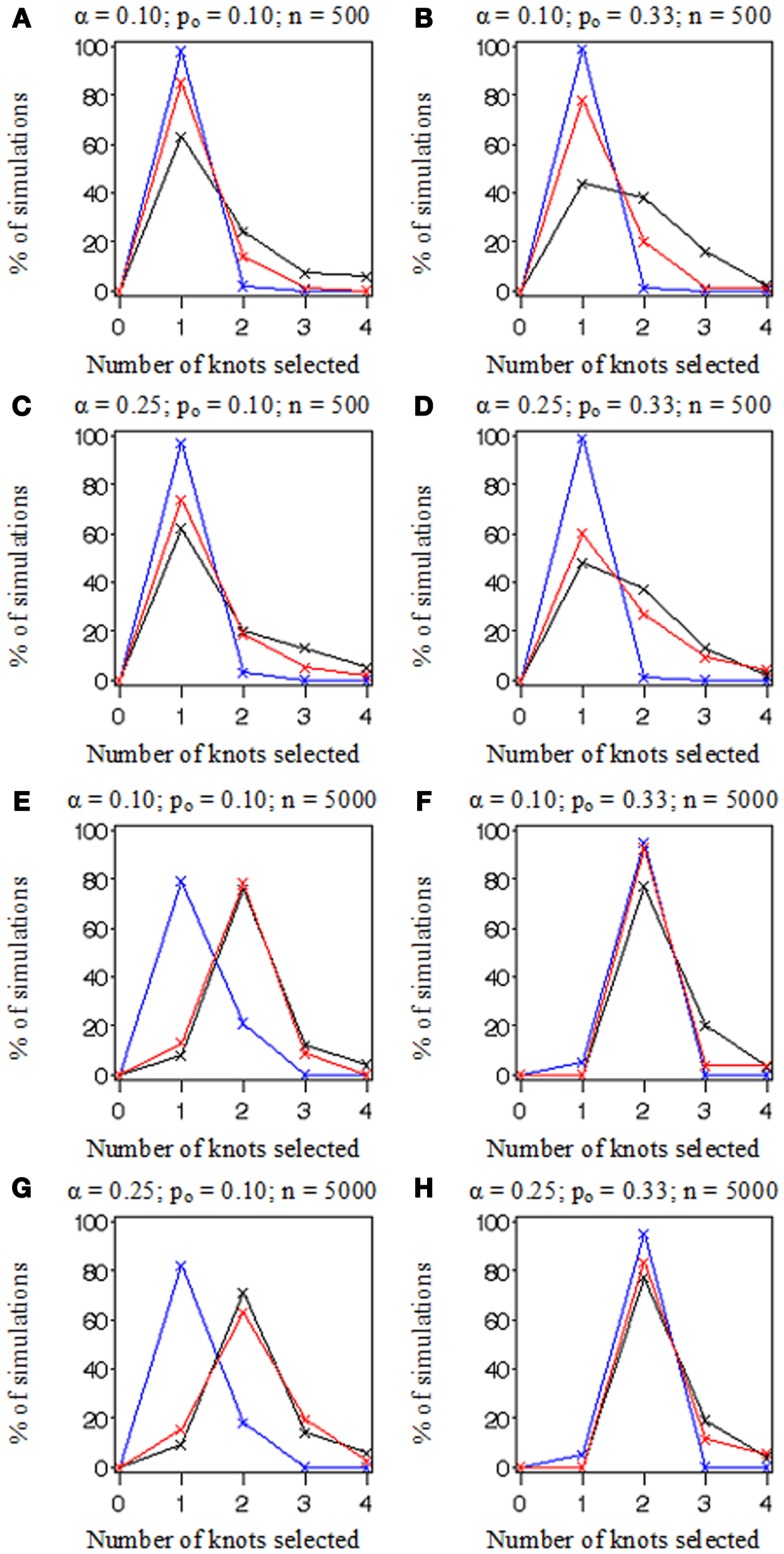
**A comparison of knot selection simulation results**. Plotted lines connect frequencies for the number of knots fitted to 100 simulated datasets by method: AIC (in black), BIC (in blue), and the parametric bootstrap MLE 2 df selection procedure (in red). Each **(A–H)** depicts results from data simulated under various conditions including the proportion of events, *p*_o_ (0.10 or 0.33) and sample size, *n* (500 or 5,000), and evaluated by our methods with two selection criterion settings, α (0.10 or 0.25).

**Figure 4:**
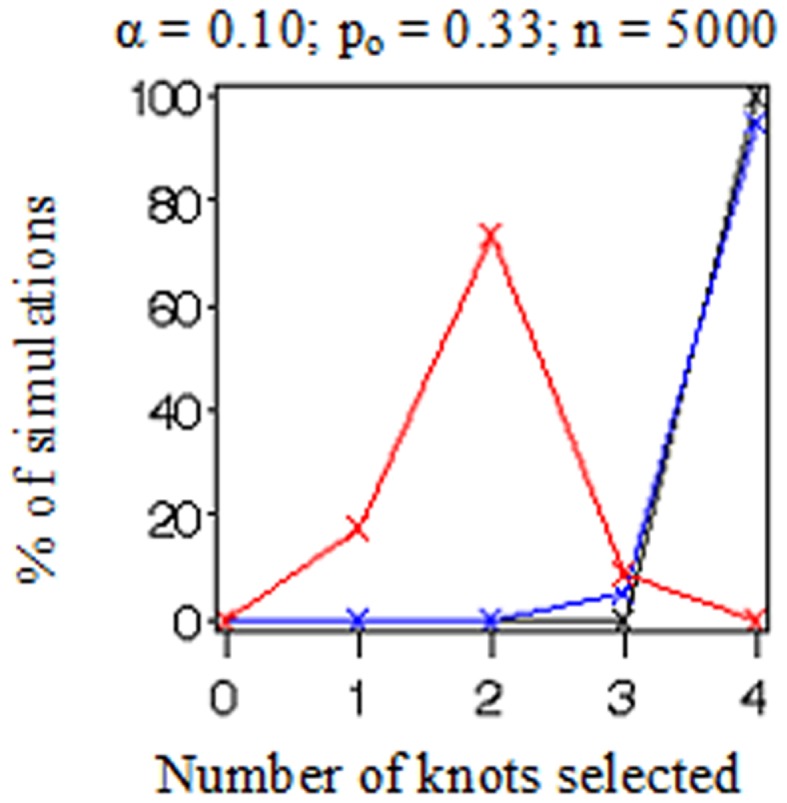
**A comparison of knot selection simulation results when sampling weights are applied**.

In Figure 3, we plotted the frequencies with which each of the number of knots (i.e., *K* = 0, 1, 2, 3, or 4) was selected as optimal from the 100 datasets simulated and evaluated under each combination of settings. Recall that the true number of knots was 2 in all simulations. Each frame has colored points representing the observed frequencies connected by colored lines representing knot selection results from our method (in red), Akaike’s Information Criterion (AIC, in black), and Bayesian Information Criterion (BIC, in blue). All these approaches were too conservative when the sample size was low (*n* = 500 in Figures 3A–D) as they tended to select models with one knot. BIC was also too conservative when the sample size was high and the proportion of events was low (*n* = 5,000, *p*_o_ = 0.10 in Figures 3E,G). Sample-weighted likelihoods from large survey samples are not on a scale by which the AIC or BIC penalties would have any effect to curb overfitting the data. We can see this result clearly in Figure 4 where our method was accurate, but somewhat imprecise while AIC and BIC methods were neither accurate nor precise. In cases where no sample weights were used, our method worked very similarly to AIC under all conditions simulated (see Figure 3). However, like AIC, our method was only accurate in selecting two knots when the sample size was relatively large (i.e., when *n* = 5,000 in Figures 3E–H). Table 1 presents statistics on the accuracy of each selection approach for correctly selecting a two-knot model. These results include pairwise Fisher’s exact tests between our parametric bootstrap MLE 2 df testing procedure and AIC or BIC. Consistent with the plots in Figures 3 and 4, AIC was the preferred approach for small sample sizes. Our approach significantly outperformed AIC only when there were sampling weights involved (*p* < 0.001) or when the sample size and prevalence was high and selection criterion was set low (*p* < 0.001). Interestingly, the only case where BIC was superior to our method was when the sample size and prevalence were high, the selection criterion was set low, and no sampling weights were involved (*p* = 0.048).

**Table 1:** **Knot selection accuracy: comparing our parametric bootstrap MLE 2 df selection procedure to AIC and BIC by how frequently they correctly select two knots in 100 datasets simulated and evaluated at each combination of sample size, prevalence, and selection criterion settings**.

Sample size (*n*)	Prevalence (*p*_o_)	Complex sample weights	Selection criterion for our test (α)	Frequency of correctly selecting 2 knots			Test 1: Ours vs. AIC, p*	Test 2: Ours vs. BIC, p*
				Our test	AIC	BIC	
500	0.10	No	0.10	13	22	2	0.136	0.006
500	0.33	No	0.10	20	38	1	0.008	<0.001
500	0.10	No	0.25	19	20	3	>0.999	<0.001
500	0.33	No	0.25	27	37	1	0.172	<0.001
5,000	0.10	No	0.10	78	76	21	0.867	<0.001
5,000	0.33	No	0.10	92	76	94	0.003	0.783
5,000	0.10	No	0.25	63	69	18	0.456	<0.001
5,000	0.33	No	0.25	83	77	93	0.377	0.048
5,000	0.33	Yes	0.10	73	0	0	<0.001	<0.001

*p-Value from two-sided Fisher’s exact test.

## Discussion

The MLE 2 df knot testing procedure for specifying the optimal number of knots generally performed well in simulations and very similar to AIC when ignoring sampling weights, as long as the sample sizes were large or when the selection criterion was set fairly high (i.e., *n* = 5000 or α = 0.25). BIC was too conservative unless both the proportion of individuals experiencing events and the sample size was large (i.e., *p*_o_ = 0.33 and *n* = 5000). Most sample sizes among complex nationally representative surveys have at least 5,000 participant records available for analyses. However, when the data are stratified and analyses are run on small subsets of the survey data, our methods may not have enough power to precisely or accurately characterize the “true” model. It is noteworthy from the simulations that neither AIC nor BIC will incur penalties sufficient enough to curb overfitting the data when complex sampling weights are applied to the likelihood functions. The weights distort the scale of the likelihood away from the penalty to the point that they no longer correct for overfitting. It is clear that the likelihood and/or the penalty terms must be normalized in some way before these AIC or BIC would work correctly in the complex sample analysis setting.

Due to computational demands and long running times associated with our MLE 2 df knot testing procedure, we conducted a relatively small simulation study and did not introduce covariates into the simulated models. More research is needed to examine how correlation structures and collinearities might influence the performance of our knot selection procedure. While we acknowledge these weaknesses, we feel that the results from the simulations are valid and characterize well important properties of this novel aspect of our modeling framework.

Our methods are intended for use on biological data in which the knot parameters have meaning with the expectation of looking for a relatively low number of knots in data where the number of observations (*n*) is much larger than the number of parameters (*q*). Given the computational intensity of applying our framework, it is not recommended for applications where *q* is close to or greater than *n*. With enough computing power, we suggest that the non-linear bases in our framework can be readily extended for fitting models with more than one non-linear predictor.

Akaike’s Information Criterion and BIC were designed for testing non-nested models. Although our MLE 2 df knot testing procedure performed well in our simulation study in comparison to AIC and BIC, it is important to note that a problem may exist for our approach to selecting the optimal number of knots, *K*. One of the foundational assumptions of the forward selection is that the model with *K* knots is nested within the model with *K* + 1 knots. As Bessaoud et al. (4) pointed out, free-knot splines in which both the number and locations of knots are estimated are non-nested, with the notable exception that the linear model (*K* = 0) is nested in all *K*-knot models. Although it is hard to imagine a well-fitted (*K* + 1)-knot model fitting any worse than a *K*-knot model, this could possibly happen since the models are not nested. Defining nested models is not a straightforward task. The following are somewhat oversimplified definitions of nested and non-nested models (47):

Two models are nested if one model can be reduced to the other model by imposing a set of linear restrictions on the parameter vector.

Two models are non-nested, either partially or strictly, if one model cannot be reduced to the other model by imposing a set of linear restrictions on the parameter vector.

These concepts provide the foundation for the asymptotic *F*-test and LR test for evaluating the contribution of sets of parameters to the overall model fit in regression analysis. Our situation with fitting free-knot parameters is more complicated than most regression applications. When a free-knot parameter is added to or removed from these models, the parameters locally fitted to construct the adjoining spline segments do not maintain their definition. If we compare two piecewise linear free-knot spline models (say, one with *K* = 1 to another with *K* = 2) fitted to the same data, we cannot say that the slope parameter to the right of the knot in the *K* = 1 model (*a*_2_) is analogous to the slope parameter (*a*_2_) between the two knots in the *K* = 2 model (note also that it is also not analogous to the parameter to the right of the second knot, *a*_3_). These models would be nested if the knot fitted in the *K* = 1 knot model was in a fixed location for the *K* = 2 knot model. However, conditioning added knots on the location of the previous knot locations undermines the properties we prize in free-knot splines.

Though our 2 df knot testing methods appeared to work well in simulations and in applications to real data (25), our approach to finding the optimal *K* from amongst non-nested candidate models may yet be improved. For instance, it would be useful to introduce penalties for coalescing knots in a fashion similar to that of Lindstrom (22). If the data model were truly improved by including jump-discontinuities, then the modeling framework should allow for this possibility. However, inducing penalties to avoid unnecessary overlapping of knots would help avoid the lethargy problem (27) and provide a more powerful approach than dropping models in which knots have coalesced, or enforcing linear constraints to ensure enough space between knots so that they might still be considered biologically meaningful, as we have done in some applications of our framework to real data.

Modeling time to event data with censored observations in complex samples is a crucial objective for our framework. We expect to extend our likelihood-based MLE methods to modeling non-linear bases in partial likelihood equations in Cox-type models (48). This will provide a foundation to begin modeling relative risks in terms of hazard ratios computed by non-linear proportional hazards regression in our framework with some modifications to the design of our MLE approach.

## Author Contributions

Scott W. Keith and David B. Allison made substantial contributions to the conception and design of this work, the analysis and interpretation of the data; drafted this work and revising it critically for important intellectual content; provided final approval of the version to be published; and agree to be accountable for all aspects of this work in ensuring that questions related to the accuracy or integrity of any part of this work are appropriately investigated and resolved.

## Conflict of Interest Statement

The authors declare that the research was conducted in the absence of any commercial or financial relationships that could be construed as a potential conflict of interest.

## Supplementary Material

The Supplementary Material for this article can be found online at http://www.frontiersin.org/Journal/10.3389/fnut.2014.00016/abstract
