# Supplementary material for: A Free-Knot Spline Modeling Framework for Piecewise Linear Logistic Regression in Complex Samples with Body Mass Index and Mortality as an Example
Source: Front Nutr. 2014 Sep 29;1:16. doi: 10.3389/fnut.2014.00016 (PMC4297674; doi:10.3389/fnut.2014.00016)
Supplement: Supplementary file 1 [file Presentation1.PDF]

Supplementary Material

2.6.3.1S. Levenberg-Marquardt adaptation to the Gauss-Newton algorithm for nonlinear LSE. Consider this nonlinear system of equations that represent our nonlinear model between a vector of outcomes, **Y**, and a function of the observed data, **X**, and parameters,

$$\mathbf{Y} = F(\theta, \mathbf{X}) + \varepsilon \;, \tag{1}$$

where **ε** is the error vector. The general approach to solving for the minimum distance between **Y** and $F\left(\hat{\boldsymbol{\theta}}, \mathbf{X}\right)$, that is, the residual distance $\mathbf{e} = \mathbf{Y} - F\left(\hat{\boldsymbol{\theta}}, \mathbf{X}\right)$, is to solve the nonlinear "normal" equations,

$$\mathbf{D}^{\mathrm{T}} F\left(\hat{\boldsymbol{\theta}}, \mathbf{X}\right) = \mathbf{D}^{\mathrm{T}}\mathbf{Y}, \tag{2}$$

where **D** represents the gradient matrix,

$$\mathbf{D} = \frac{\partial F\left(\hat{\boldsymbol{\theta}}, \mathbf{X}\right)}{\partial \hat{\boldsymbol{\theta}}}. \tag{3}$$

Note that, in practice, we cannot actually calculate **D** because the derivatives at the knot locations do not exist. Instead, we used finite difference approximations to **D**.

A closed form solution to (2) generally will not exist, so we try to find a solution by an iterative process beginning with some starting value for the values, $\hat{\boldsymbol{\theta}}_{\text{old}}$, and continuing to update $\hat{\boldsymbol{\theta}}_{\text{old}}$ to $\hat{\boldsymbol{\theta}}_{\text{new}}$ until $\mathbf{e}^{\mathrm{T}}\mathbf{e}$, the residual sum of squares (SSE), shows no major improvement after reiterating,

$$\mathrm{SSE}(\hat{\boldsymbol{\theta}}_{\text{new}}) = \mathrm{SSE}(\hat{\boldsymbol{\theta}}_{\text{old}} + k\Delta) < \mathrm{SSE}(\hat{\boldsymbol{\theta}}_{\text{old}}), \tag{4}$$

where **D** represents the next "step," and *k* is a coefficient that can be adjusted to control the size of the step.

For this LSE approach to numerically solving the piecewise linear function optimization problem, SAS software offers several popular iterative algorithms. We chose the Levenber-Marquardt updating formula (Levenberg, 1944;Marquardt, 1963) defined as follows:

$$\boldsymbol{\Delta} = \left(\mathbf{D}^{\mathrm{T}}\mathbf{D} + \lambda \operatorname{diag}\left(\mathbf{D}^{\mathrm{T}}\mathbf{D}\right)\right)^{-} \mathbf{D'e}. \tag{5}$$

This method is a compromise between the Gauss-Newton and steepest descent ($\boldsymbol{\Delta} = \mathbf{D'e}$) methods (Marquardt, 1963) affected by adjusting the magnitude of λ. Some have suggested that for estimating free-knot parameter locations, the Levenberg-Marquardt method increases the chance of finding the global optimum (Lindstrom, 1999).

Supplemental Tables

Table S1. The LSE approach to the 2 degree of freedom knot testing procedure

The null hypothesis that the "true" model has K_{null} knots versus an alternative of $K_{alt.}$ can be specified for model parameters ($\boldsymbol{\theta}$; including linear and nonlinear free parameters as expressed in (11)) fitted to a dataset having binary outcome (**Y**), potentially nonlinear continuous predictor (**X**), covariates (**Z**), and sample weights (**W**) by these algorithm specifications:

Step 1: Set: $K_{null} = 0$, $K_{alt.} = K_{null} + 1$;
Step 2: Input: **Y**, **X**, **Z**, **W;**
Step 3: Initialize: $\boldsymbol{\theta}^0_{null}, \boldsymbol{\theta}^0_{alt.}$;
Step 4: Minimize: $\arg\min(\mathrm{SSE}(\boldsymbol{\theta}_{null})) \rightarrow \hat{\boldsymbol{\theta}}_{null}$, start = $\boldsymbol{\theta}^0_{null}$;
  Compute: $\mathrm{SSE}(\hat{\boldsymbol{\theta}}_{null})$;
Step 5: Minimize: arg min(objective = $\mathrm{SSE}(\boldsymbol{\theta}_{alt.}) \rightarrow \hat{\boldsymbol{\theta}}_{alt.}$, start = $\boldsymbol{\theta}^0_{alt.}$;
  Compute: $\mathrm{SSE}(\hat{\boldsymbol{\theta}}_{alt.})$;
Step 6: Compute: $F = \dfrac{(\mathrm{SSE}_{null} - \mathrm{SSE}_{alt.})/2}{\mathrm{SSE}_{alt.}/\mathrm{df}_{alt.}}$;
Step 7: Parametric Bootstrap: for $j = 1$ to D_1 do
  Generate $\mathbf{Y}^{rep}_j$ by drawing a random binary outcome for each subject, $i = 1,\ldots,N$, from Bernoulli($p_i | \hat{\boldsymbol{\theta}}_{null}$);
  Repeat Steps 2 through 6 replacing **Y** with $\mathbf{Y}^{rep}_j$;
  Compute: F^{rep}_j of F under H_o: $\boldsymbol{\theta} = \hat{\boldsymbol{\theta}}_{null}$;
  End do;
Step 9: Compute: $p_{boot} = \dfrac{1}{D_1 + 1}\left(1 + \sum_{j=1}^{D_1} \mathrm{I}\{F^{rep}_j \le F\}\right)$;
Step 10: Select the model: If $p_{boot} \le \alpha$ and $K_{null} \le 2$ then do
  Set $K_{null} = K_{null} + 1$, $K_{alt.} = K_{alt.} + 1$;
  Repeat Steps 2 through 9;
  End do;
  Else if $p_{boot} \le \alpha$ and $K_{null} = 3$ then do
  $K = 4$;
  $\hat{\boldsymbol{\theta}} = \hat{\boldsymbol{\theta}}_{alt.}$;
  End do;
  Else do;
  $K = K_{null}$;
  $\hat{\boldsymbol{\theta}} = \hat{\boldsymbol{\theta}}_{null}$;
  End do;
Step 11: Compute: $\hat{\boldsymbol{\theta}}^{PLS}$ from $\hat{\boldsymbol{\theta}}$ where B-spline parameter elements have been linearly transformed to piecewise linear slope parameters;

Table S2. The MLE approach to the 2 degree of freedom knot testing procedure.

For the MLE, we adopted a similar approach, but with a likelihood ratio (*LR*) test statistic in place of the F-ratio statistics. The algorithm is specified as follows:

Step 1: Set: $K_{\text{null}} = 0$, $K_{\text{alt.}} = K_{\text{null}} + 1$;

Step 2: Input: **Y**, **X**, **Z**, **W**;

Step 3: Initialize: $\boldsymbol{\theta}^0_{\text{null}}, \boldsymbol{\theta}^0_{\text{alt.}}$;

Step 4: Minimize: arg min(objective = -ln[L($\boldsymbol{\theta}_{\text{null}}$)])→ $\hat{\boldsymbol{\theta}}_{\text{null}}$, start = $\boldsymbol{\theta}^0_{\text{null}}$;

Compute: -ln[L($\hat{\boldsymbol{\theta}}_{\text{null}}$)];

Step 5: Minimize: arg min(objective = -ln[L($\boldsymbol{\theta}_{\text{alt.}}$)])→ $\hat{\boldsymbol{\theta}}_{\text{alt.}}$, start = $\boldsymbol{\theta}^0_{\text{alt.}}$;

Compute: -ln[L($\hat{\boldsymbol{\theta}}_{\text{alt.}}$)];

Step 6: Compute: $LR = \dfrac{-\ln \mathrm{L}(\hat{\boldsymbol{\theta}}_{\text{null}})}{-\ln \mathrm{L}(\hat{\boldsymbol{\theta}}_{\text{alt.}})}$;

Step 7: Parametric Bootstrap: for $j = 1$ to D_1 do

Generate $\mathbf{Y}_j^{\text{rep}}$ by drawing a random binary outcome for each subject, $i = 1,\ldots,N$, from Bernoulli($p_i|\hat{\boldsymbol{\theta}}_{\text{null}}$);

Repeat Steps 2 through 6 replacing **Y** with $\mathbf{Y}_j^{\text{rep}}$;

Compute: LR_j^{rep} of *LR* under H_o: $\boldsymbol{\theta} = \hat{\boldsymbol{\theta}}_{\text{null}}$;

End do;

Step 9: Compute: $p_{\text{boot}} = \dfrac{1}{D_1 + 1}\left(1 + \sum_{j=1}^{D_1} \mathrm{I}\{LR_j^{\text{rep}} \le LR\}\right)$;

Step 10: Select the model: If $p_{\text{boot}} \le \alpha$ and $K_{\text{null}} \le 2$ then do

Set $K_{\text{null}} = K_{\text{null}} + 1$, $K_{\text{alt.}} = K_{\text{alt.}} + 1$;

Repeat Steps 2 through 9;

End do;

Else if $p_{\text{boot}} \le \alpha$ and $K_{\text{null}} = 3$ then do

K = 4;

$\hat{\boldsymbol{\theta}} = \hat{\boldsymbol{\theta}}_{alt.}$;

End do;

Else do;

$K = K_{\text{null}}$;

$\hat{\boldsymbol{\theta}} = \hat{\boldsymbol{\theta}}_{\text{null}}$;

End do;

Step 11: Compute: $\hat{\boldsymbol{\theta}}^{\text{PLS}}$ from $\hat{\boldsymbol{\theta}}$ where B-spline parameter elements have been linearly transformed to piecewise linear slope parameters;

Table S3. A nonparametric bootstrap procedure algorithm for calculating standard errors and 95% confidence intervals for parameter estimates by the LSE approach.

Step 1: Input: **Y**, **X**, **Z**, **W**
Step 2: Nonparametric bootstrap: for $j = 1$ to D_2 do
  for $h = 1$ to H do
  resample with replacement $m_h = n_h - 1$ PSUs from stratum h;
  rescale sample weights;
  End do;
  Minimize: arg min(objective = SSE($\boldsymbol{\theta}$)→ $\hat{\boldsymbol{\theta}}_j$, start = $\hat{\boldsymbol{\theta}}$;
  Compute: $\hat{\boldsymbol{\theta}}_j^{\text{PLS}}$ where B-spline parameter elements have been linearly transformed to a piecewise linear slope parameters;
  End do;
Step 3: Let $\boldsymbol{\Lambda}_i^{\text{T}}$ represent the vector transopose of the ith row of the matrix
  $\boldsymbol{\Lambda} = \left[\hat{\boldsymbol{\theta}}_1^{\text{PLS}} \quad \ldots \quad \hat{\boldsymbol{\theta}}_{\text{D}_2}^{\text{PLS}}\right]_{p\times\text{N}}$;
Step 4: Compute SE and 95% CI for each model parameter, i: for $i = 1$ to p do
  Compute: $\bar{\hat{\theta}}_i^{\text{PLS}} = \frac{1}{\text{D}_2}\mathbf{1}^{\text{T}}\boldsymbol{\Lambda}_i^{\text{T}}$;
  Compute: $\hat{\sigma}_i^* = \sqrt{\frac{1}{\text{D}_2 - 1}\left(\boldsymbol{\Lambda}_i^{\text{T}} - \bar{\hat{\theta}}_i^{\text{PLS}}\right)^{\text{T}}\left(\boldsymbol{\Lambda}_i^{\text{T}} - \bar{\hat{\theta}}_i^{\text{PLS}}\right)}$;
  Sort: $\boldsymbol{\Lambda}_i^{\text{T}}$ in ascending order;
  Compute: $\boldsymbol{T}_i^* = \left\{T_{i(1)}^*, \ldots, T_{i(\text{D}_2)}^*\right\}$ from $\boldsymbol{\Lambda}_i^{\text{T}}$
  Compute: P($T_i{}^*_{\text{(lower critical)}} < T_i < T_i{}^*_{\text{(upper critical)}}$) ≥ 0.95 with equal probability in either tail;
  Compute: 95% CI for $\boldsymbol{\theta}_i^{\text{PLS}}$ from $T_i{}^*_{\text{(lower critical)}}$ and $T_i{}^*_{\text{(upper critical)}}$;
  End do;

**Table S4. A nonparametric bootstrap procedure algorithm for calculating standard errors and 95% confidence intervals for parameter estimates by the MLE approach.**
For the MLE, we decided to implement the more conservative percentile method of calculating the 95% confidence intervals and do all optimizations with the B-spline basis.<br>Step 1: Input: **Y**, **X**, **Z**, **W**<br>Step 2: Nonparametric bootstrap: for $j = 1$ to D_2 do<br>for $h = 1$ to H do<br>resample with replacement $m_h = n_h - 1$ PSUs from stratum h;<br>rescale sample weights;<br>End do;<br>Minimize: arg min(objective = -ln[L($\boldsymbol{\theta}$)])→ $\hat{\boldsymbol{\theta}}_j$, start = $\hat{\boldsymbol{\theta}}$;<br>Compute: $\hat{\boldsymbol{\theta}}_j^{\mathrm{PLS}}$ where B-spline parameter elements have been linearly transformed to a piecewise linear slope parameters;<br>End do;<br>Step 3: Let $\boldsymbol{\Lambda}_i^{\mathrm{T}}$ represent the vector transopose of the ith row of the matrix<br>$\boldsymbol{\Lambda} = \left[\hat{\boldsymbol{\theta}}_1^{\mathrm{PLS}} \quad \dots \quad \hat{\boldsymbol{\theta}}_{\mathrm{D}_2}^{\mathrm{PLS}}\right]_{p\times\mathrm{N}}$;<br>Step 4: Compute SE and 95% CI for each model parameter, i: for $i = 1$ to p do<br>Compute: $\bar{\hat{\theta}}_i^{\mathrm{PLS}} = \frac{1}{\mathrm{D}_2}\mathbf{1}^{\mathrm{T}}\boldsymbol{\Lambda}_i^{\mathrm{T}}$;<br>Compute: $\hat{\sigma}_i^* = \sqrt{\frac{1}{\mathrm{D}_2 - 1}\left(\boldsymbol{\Lambda}_i^{\mathrm{T}} - \bar{\hat{\theta}}_i^{\mathrm{PLS}}\right)^{\mathrm{T}}\left(\boldsymbol{\Lambda}_i^{\mathrm{T}} - \bar{\hat{\theta}}_i^{\mathrm{PLS}}\right)}$;<br>Sort: $\boldsymbol{\Lambda}_i^{\mathrm{T}}$ in ascending order;<br>Set lower bound for the 95% CI of $\boldsymbol{\theta}_i^{\mathrm{PLS}}$ to the 2.5th percentile of $\boldsymbol{\Lambda}_i^{\mathrm{T}}$;<br>Set upper bound for the 95% CI of $\boldsymbol{\theta}_i^{\mathrm{PLS}}$ to the 97.5th percentile of $\boldsymbol{\Lambda}_i^{\mathrm{T}}$;<br>End do;

References for Supplemental Materials

Levenberg, K. (1944). A method for the solution of certain problems in least squares. *The Quartery of Applied Mathematics* 2**,** 164-168.

Lindstrom, M.J. (1999). Penalized estimation of free-knot splines. *Journal of Computational and Graphical Statistics* 8**,** 333-352.

Marquardt, D. (1963). An algorithm for least-squares estimation of nonlinear parameters. *SIAM J Appl Math* 11**,** 431-441.
